# Dietary xenobiotics, (poly)phenols and fibers: Exploring associations with gut microbiota in socially vulnerable individuals

**DOI:** 10.3389/fnut.2022.1000829

**Published:** 2022-10-12

**Authors:** Aida Zapico, Silvia Arboleya, Sergio Ruiz-Saavedra, María Gómez-Martín, Nuria Salazar, Alicja M. Nogacka, Miguel Gueimonde, Clara G. de los Reyes-Gavilán, Sonia González

**Affiliations:** ^1^Department of Functional Biology, University of Oviedo, Oviedo, Spain; ^2^Diet Microbiota and Health Group, Instituto de Investigación Sanitaria del Principado de Asturias (ISPA), Oviedo, Spain; ^3^Department of Microbiology and Biochemistry of Dairy Products, Instituto de Productos Lácteos de Asturias (IPLA-CSIC), Villaviciosa, Spain

**Keywords:** xenobiotic, microbiota, sustainable diet, fiber, Mediterranean diet, meat

## Abstract

**Objectives:**

Although xenobiotics derived from food processing may cause modifications in the composition of the gut microbiota (GM) evidence is scarce. The aim of this study is to evaluate the impact of potential dietary carcinogens as heterocyclic amines (HAs), polycyclic aromatic hydrocarbons (PAHs), nitrates, nitrites, nitroso compounds and acrylamide, in combination to fibers (poly)phenols on the GM composition in a group of materially deprived subjects.

**Study design:**

Transversal observational study in a sample of 19 subjects recipients of Red Cross food aid. Dietary information was recorded by means of 3 non-consecutive 24 h recalls. Questions focused on the type of cooking and the extent of cooking and roasting were included. Information on potential carcinogens was mainly obtained from the European Prospective Investigation into Cancer and Nutrition (EPIC) and Computerized Heterocyclic Amines Resource for Research in Epidemiology of Disease (CHARRED) Carcinogen Databases. Microbial composition was determined by 16S ribosomal RNA gene sequencing in fecal samples.

**Results:**

Higher levels of Lachnospiraceae and Eggerthellaceae families were found in individuals consuming less than 50 ng/day of 2-amino-3,8 dimethylimidazo (4,5,f) quinoxaline (MeIQx) (considered as lower risk dose for colorectal adenoma) while those consuming more than 40 ng/day of 2-amino-1-methyl-6-phenylimidazo (4,5,b) pyridine (PhIP) (higher risk for colorectal adenoma) showed lower relative abundance of Muribaculaceae and greater presence of Streptococcaceae and *Eubacterium coprostanoligenes* group.

**Conclusion:**

The associations identified between diet and processing by-products on GM in this study could be used as potential targets for the designing of dietary interventions tailored to this collective.

## Introduction

Gut microbiota (GM) is the set of microorganisms, including bacteria, archaea, viruses, and some unicellular eukaryotes, which inhabit the digestive tract, the colon being the area most densely populated (1). In adults living in developed countries, the most abundant intestinal microorganisms are bacteria belonging to the phyla Bacteroidota, Bacillota (former Firmicutes), Actinobacteriota, Pseudomonadota (former Proteobacteria), and Verrucomicrobiota in a lesser proportion (2–4). In the last years, there was increasing scientific evidence supporting the critical role of the GM in the maintenance of gut homeostasis and in the prevention of different non-communicable diseases, particularly metabolism-related conditions, and several types of cancers (4). From environmental factors, diet plays a fundamental role in shaping the composition and activity of the GM and, thus, determines the inter-relationship between the gut microbiome and the host (5). In humans, the transition from the ancestral diet to the current westernized pattern, represented by a high presence of fats, sugars, animal proteins and processed foods, has shown to impact on GM composition and activity, by means of the reduction in the abundance of certain microorganisms such as *Prevotella* or *Xylanobacter* with capacity to degrade cellular wall components from plants as cellulose and xylan (6) and by a drastic decrease in microbial diversity (6–8). This dietary change may be particularly striking in socially disadvantaged groups where an increase in consumption of processed foods high in fat, sugar or salt has been detected (9), as well as a decrease in the intake of fresh products, such as fruit and vegetables or fish (10). Apart from the nutritional aspects, modern diets have led to an increased intake of processed food. Xenobiotic is a general term used to define “*chemical substances that are foreign to animal life*” including vegetable derived compounds, drugs or additives (11). Within this broad term, the International Agency of Research in Cancer (IARC) provides a particular category for those substances which exhibit demonstrated cytotoxic and genotoxic character (12). In this sense, heterocyclic amines (HAs) and polycyclic aromatic hydrocarbons (PAHs) are generated during high-temperature cooking by grilling, barbecuing, or frying processes, being their concentration in the food directly proportional to the exposure time and temperature (13, 14). Some authors have proposed that these substances may lead to modifications of the GM composition and functions, with influence in host homeostasis (15, 16), although scientific evidence in this field is still scarce (17). The GM also plays a pivotal role as producer and transformer of xenobiotics to activated derivatives, and/or as attenuator of the toxic action of these compounds by diverse mechanisms (18). Some lactic acid bacteria and other microorganisms can bind or metabolize xenobiotics, contributing either to their sequestration and excretion in feces, or to their transformation into less toxic compounds (19, 20) whereas sometimes the GM can metabolize xenobiotics and transform them into derived molecules with enhanced toxicity (17). Some gastrointestinal microbes can generate toxic compounds themselves, as is the case of the colibactin produced by *Escherichia coli* group B (21) or enterotoxins formed by *Bacteroides fragilis*, among others (22). The modification of the toxicity of some dietary xenobiotics can also occur through interactions between the GM and host-detoxification mechanisms. This mainly involves impairment of the activity of hepatic enzymes from the cytochrome P450 complex that participates in the hepatic phase I of detoxification (23) and the reactivation of deactivated glucuronic acid-conjugated compounds during the phase II of detoxification in the liver, by the activity of microbial β-glucuronidases harbored by some intestinal enterobacteria and by members of *Clostridium* and *Bacteroides* genera (24). It is also worth mentioning that some members of the GM can metabolize dietary (poly)phenols, promoting the increase of their biological health benefits, as it is the case of the transformation of ellagitannins into urolithins by members of *Clostridium leptum* group and *Bacteroides/Prevotella* (25) and the conversion of lignans into enterolignans, process in which can participate some *Clostridium*, *Bacteroide*s, *Peptostreptococcus* and *Eggerthella* strains, among others (26). Dietary fiber can also act as a sequestrating agent of some toxic dietary compounds, decreasing intestinal toxicity (27). Based on this evidence, the main objective of this pilot study was to analyze the associations between diet and GM, with special emphasis on bioactive compounds and xenobiotics derived from food processing, in a group of materially deprived subjects with a diet rich in processed foods.

## Materials and methods

### Participants and study design

The MESAS (Economic, Healthy, and Sustainable Menus) pilot project corresponds to an educational and dietary program in deprived human groups addressed to recipients of food aid from the Red Cross of Asturias (North of Spain). The aid received usually consists of basic non-perishable foodstuffs packs. Thus, in order to offer didactic and dietary tools adapted to these individuals and to facilitate the acquisition of fresh food for the achievement of a balanced and healthy diet, the aim of the MESAS project is to identify the main dietary targets in this group. The data presented in this work are relative to the basal status of the sample studied in which twenty adult subjects with non- declared pathologies were randomly selected among those receiving food aid from Alimerka Foundation provided by the non-profit organization Spanish Red Cross. Inclusion criteria were not to be diagnosed with any chronic condition and not to have consumed antibiotics in the last month. The subjects participating in the aid program were randomly recruited and informed of the objectives of the study, being those interested in participating invited to a personal interview in which the purpose of the project and the required involvement were explained. The volunteers who agreed to participate signed an informed consent form. From the population sample, only those for which both dietary and GM information was available were included in the present study (*n* = 19).

The whole procedure and methodology of this project was approved by the Ethics Committee of the Hospital Universitario Central de Asturias (CEImPA2021.307). The procedures were performed in accordance with the fundamental principles set out in the Declaration of Helsinki, the Oviedo Bioethics Convention, and the Council of Europe Convention on Human Rights and Biomedicine, as well as in Spanish legislation on Bioethics. Directive 95/46/EC of the European Parliament and the Council of October 1995, on the protection of individuals regarding the processing of personal data and on the free movement of such data was strictly followed.

### Nutritional assessment

Information regarding the dietary intake of the participants was collected by means of a one unique personalized interview, of no more than 30 min of duration, through three non-consecutive 24 h recalls. At the same interview, participants were scheduled for blood collection and were given the fecal sample collection bottle. Specific questions about cooking habits (boiled, fried, grilled, baked/broiled, or barbecued) and the degree of doneness or toasting in the case of meats, fried potatoes, or toasted bread (undercooked, medium, well-done, very well-done) were included. In order to standardize this point, photographs of the different cooking temperatures, in which the degree of browning increased progressively, were developed specifically for this study. Additionally, complementary questions such as which part of the food was consumed (breast or thigh in the case of chicken) or the possible consumption and/or cooking of the skin (cooking with skin and eating the skin; cooking with skin, but not consuming it; and cooking without skin) were incorporated to improve the quality of the information. The classification of the food into groups was carried out according to the Centre for Higher Education in Nutrition and Dietetics (CESNID) criteria. Food composition tables of CESNID (28) and the United States Department of Agriculture (USDA) (29) were used to transform food consumption into energy and macronutrients intake. The fiber and phenolic content of the foods was extracted from Marlett and Cheung tables (30) and the Phenol Explorer 3.6 (31). For each dietary compound, the five major food sources were identified.

#### Nutritional assessment of xenobiotics derived from food processing

The nutritional analysis of the sample was carried out based on food consumption per individual. Information on HAs, PAHs, nitrates, and nitrites was mainly obtained from the European Prospective Investigation into Cancer and Nutrition (EPIC) Carcinogen Database (32). The EPIC database compiles information from 139 references regarding the content of these compounds per 100 g of food in more than 200 food items. The food composition table is classified according to the preservation method, cooking method, degree of browning, and temperature (32). For those foods or culinary preparations not included in the EPIC database, information was completed with the Computerized Heterocyclic Amines Resource for Research in Epidemiology of Disease (CHARRED) database (33) in the case of HAs and benzo (a) pyrene (B(a)P), and the European Food Safety Authority (EFSA) data in the case of nitrates (34). Acrylamide content was provided by the U.S. Food and Drug Administration (FDA) composition tables (35) and other external reference sources have been used when necessary for acrylamide (36–38), HAs (39), total PAHs (40) and nitrosamines (41–44).

### Anthropometrical and biochemical determinations

Height (m) and weight (kg) were taken by standardized protocols (45) and Body Mass Index (BMI) was calculated using the formula: weight/(height)^2^. Subjects were classified as normal weighted (18.5–24.9 kg/m^2^), overweighted (25.0–29.9 kg/m^2^), and obese (≥ 30.0 kg/m^2^), based on the Spanish Society for the Study of Obesity (SEEDO) criteria (46). The percentage of body fat was estimated through bioelectrical impedance in a calibrated TANITA equipment (Tanita Corporation of America, Inc., Arlington Heights, IL, USA). Waist and hip circumferences were measured with an inelastic and extensible tape according to standard criteria (47) and waist-hip ratio was calculated as the ratio of waist circumference over the hip circumference.

Fasting blood samples were drawn by venipuncture and collected in separate tubes for serum and plasma. Samples were kept on ice and centrifuged (1,000 x g, 15 min) within 2–4 h after collection. Plasma and serum aliquots were stored at –20°C until analysis were performed. From blood samples, biochemical analysis of fasting plasma glucose, cholesterol, high- and low-density lipoproteins (HDL and LDL) and triglycerides were determined by using an automated biochemical autoanalyzer in external laboratories.

### Fecal microbiota analysis

Fecal samples were collected within ± 24 h of blood collection in sterile containers supplied to each volunteer along with the instructions for sample collection. Samples were frozen within a period no longer than two hours from deposition and stored at −20°C until analysis. At lab, fecal samples were weighted, diluted 1/10 (p/v) in sterile PBS solution and homogenized at full speed (260 rpm) in a LabBlender 400 stomacher (Seward Medical, London, UK) for 3 min. Samples were centrifuged (13 000 rpm, 15 min, 4°C) and then, supernatant, and bacterial pellet were separated. From the pellet obtained, DNA was extracted in accordance with the Q Protocol for DNA extraction defined by the International Human Microbiome Standards Consortium (48) using QIAamp Fast DNA Stool Mini Kit (Qiagen, Sussex, UK). Quantification of extracted/purified DNA and 260/280 ratio was performed using Take3 Micro-Volume plate and Gen5 microplate reader (BioTek Instrument Inc., Winooski, VT, USA). DNA was finally kept frozen at –80°C until analysis.

Variable region V3-V4 of bacterial 16S rRNA genes present in each fecal community was amplified by PCR and the resulting amplicons were sequenced on an Illumina NovaSeq 6000 platform instrument. Following sequencing of the library, the obtained individual sequence reads were filtered to remove low quality sequences. All Illumina quality-approved, trimmed, and filtered data were exported, and the information was integrated in order to generate *de novo* 16S rRNA Operational Taxonomic Units (OTUs) with ≥97% sequence homology using Uparse software (Uparse v7.0.1090) (49). All reads were classified to the lowest possible taxonomic rank using Quantitative Insights Into Microbial Ecology (QIIME) and a reference dataset from the SILVA 138 database (50). The whole procedure of sequencing and annotation was undergone at Novogene Bioinformatics Technology Co., Ltd.

#### Statistical analysis

Results were analyzed using the IBM SPSS software version 25.0 (IBM SPSS, Inc., Chicago, IL, USA). Goodness of fit to the normal distribution was checked by means of the Kolmogorov-Smirnov test. As normality of the variables was not achieved, non-parametric tests were used. Overall, categorical variables were summarized as number and percentage (n (%)) and continuous variables as median and percentiles 25 and 75 (P_25_ – P_75_). Spearman correlation and stepwise regression analysis (adjusted for BMI, age and energy intake) were conducted. Heatmaps were generated using ClustVis web tool (51) and logarithmic Linear Discriminant Analysis (LDA) Effect Size (LEfSe) within the Galaxy web application (52). LefSe graphs were created for the xenobiotic compounds, derived from food processing, showing statistically significant results in Spearman correlations and stepwise regression analysis conducted and for which a risk threshold was available in the literature.

## Results

### General characteristics of the sample

A general description of the main general lifestyle, anthropometric and clinical history characteristics of the population under study is shown in Table 1. The volunteers were mostly women under 50 years living in a family unit of 3 or 4 members. Regarding lifestyle and anthropometric characteristics, about half of the sample reported less than 6 hours of sleep per day and a BMI ≥ 30 kg/m^2^.

**TABLE 1:** General characteristics and clinical history of the sample population.

Characteristics	*N* = 19
***General***	
**Age (years)**	41 (32 – 51)
**Gender**	
Female	16 (84)
**Educational level**	
Primary	4 (21)
Secondary	5 (26)
Technical	7 (37)
University	3 (16)
**Family members (n)**	
1 – 2	7 (37)
3 – 4	10 (53)
≥ 5	2 (11)
***Lifestyle***	
Sleep (hours/day)	5.5 (5.0 – 7.5)
Physical activity (walking min/day)	60.0 (21.4 – 90.0)
**Smoking status**	
Current smoker	5 (26)
Occasional alcohol consumption	6 (32)
***Anthropometric***	
**BMI (kg/m^2^)**	27.85 (22.39 – 36.00)
Underweight (≤18.5)	1 (5)
Normal weight (18.5–24.9)	6 (32)
Overweight (25.0–29.9)	3 (16)
Obese (≥30.0)	9 (47)
Total body fat (%)	45 (29 - 53)
Waist-hip ratio	0.85 (0.80 – 0.91)
***Clinical history***	
**Chronic conditions**	
Respiratory diseases	9 (47)
Stool frequency (times/week)	7.0 (5.0 – 10.0)
**Stool consistency**	
Liquid	1 (5)
Soft	13 (68)
Hard	5 (26)
Presence of occasional bleeding	2 (11)
***Biochemical parameters***	
Glucose (mg/dL)	90.0 (83.0 – 93.0)
Triglycerides (mg/dL)	130.0 (69.0 – 162.0)
Total cholesterol (mg/dL)	205.0 (193.0 – 226.0)
LDL (mg/dL)	123.0 (108.0 – 137.0)
HDL (mg/dL)	54.0 (46.0 – 67.0)
Total cholesterol/HDL ratio	3.7 (2.9 – 4.5)
LDL/HDL ratio	2.2 (1.8 – 3.0)

Data is expressed as median (P_25_ – P_75_) or as number of participants (*n* (%)) for continuous and categorical variables, respectively.

BMI, body mass index; LDL, low-density lipoprotein; HDL, high-density lipoprotein.

### Dietary pattern: Food groups, xenobiotics, (poly)phenols, and fibers

A brief description of the food intake in the sample is presented in Table 2. The median energy daily intake is approximately 1,500 kcal, being meat and derivates above the maximum recommended level of 100 g/day. Also, the daily intake of red meat and processed meats were below the upper limits of 100 g/day and 50 g/day recommended, respectively, by the World Cancer Research Fund International (WCRF) (53). No ethanol consumption has been reported in the sample.

**TABLE 2:** Description of the energy and food groups intake in the sample of study.

Characteristics	*N* = 19
Energy (kcal/day)	1510.35 (1201.45 - 1667.14)
***Food groups (g/day)***	
Cereals and cereals products	121.53 (60.70 - 151.63)
Whole grain cereals	0.00 (0.00 - 5.90)
Milk and dairy products	187.50 (92.00 – 300.00)
Meat and meat products	110.00 (93.99 - 177.04)
White meat	53.58 (33.30 - 100.38)
Red meat	27.08 (5.90 - 63.33)
Processed meat	13.33 (6.67 - 31.33)
Eggs	52.67 (24.43 - 69.93)
Fish	25.33 (0.00 – 53.00)
Seafood	0.00 (0.00 – 20.00)
Oils and fats	16.73 (11.00 - 23.99)
Vegetables	104.96 (43.37 – 190.00)
Legumes	11.67 (2.50 - 33.33)
Potatoes and tubers	43.18 (8.33 – 59.00)
Fruits	116.56 (59.10 - 170.35)
Nuts and seeds	0.00 (0.00 – 10.00)
Sugar and sweets	12.33 (7.00 - 23.33)
Snacks	0.00 (0.00 – 0.00)
Sauces and condiments	18.50 (2.50 - 30.83)
Other foods	0.00 (0.00 - 113.33)
Non-alcoholic beverages (mL/day)	300.00 (143.33 – 400.00)
Alcoholic beverages (mL/day)	(0.00 – 0.00)

Data is expressed as median (P_25_ – P_75_).

The major food sources of xenobiotics, fibers and (poly)phenols in the sample are shown in Table 3. The HA 2-amino-3,8 dimethylimidazol (4,5, f) quinoxaline (MeIQx) is provided by chicken, breast, pork and minced meat. Insoluble dietary fiber derives from white bread, potato, and pasta, among others. In addition, coffee has been identified as one of the major contributors to (poly)phenol and phenolic acids consumption (Table 3).

**TABLE 3:** Top dietary sources of xenobiotics (poly)phenols and fibers in the sample of study.

Compound	Intake	Dietary sources	(%)
***Xenobiotic compounds***			
**Heterocyclic amines (ng/day)**			
MeIQx	40.62 (3.54 – 65.45)	Chicken, breast, skinless; pork loin; minced meat, seasoned, for stuffing; beef, topside; cod, fresh	74
DiMeIQx	1.60 (0.00 – 23.35)	Chicken, breast, skinless; minced meat, seasoned, for stuffing; beef, topside; lamb, shoulder, lean and fat; pork, chops, lean and fat	58
PhIP	2.95 (0.00 – 298.97)	Chicken, breast, skinless; minced meat, seasoned, for stuffing; beef, topside; chicken, thigh, skinless; lamb, rib/chop, full fat	62
**Polycyclic aromatic hydrocarbons (μg/day)**			
B(a)P	0.04 (0.02 – 0.06)	Sunflower oil; yogurt, whole; olive oil, virgin; banana; apple, unpeeled	42
DiB(a)A	0.00 (0.00 – 0.01)	Chicken egg, whole; tea, infusion; chocolate, with milk; white bread, stick; coffee, infusion	86
Total PAH	0.70 (0.50 – 1.35)	Pizza, tomato and cheese, baked; wheat flour; pasta; white bread, stick; potato	64
**Nitrates, nitrites and nitroso compounds**			
Nitrates (mg/day)	38.19 (20.29 – 90.45)	Lettuce; potato; onion; green bean; carrot	79
Nitrites (mg/day)	0.63 (0.31 – 1.06)	Cooked ham, extra; chicken egg, whole; potato; sausage, Frankfurt type; cured ham, lean and fat	76
NDMA (μg/day)	0.03 (0.02 – 0.09)	Cooked ham, extra; chorizo, category w/s; Manchego cheese, semi-matured; black pudding; cured ham, lean and fat	79
NPIP (μg/day)	0.02 (0.01 – 0.05)	Cooked ham, extra; chorizo, category w/s; black pudding; cured ham, lean and fat; sausage, Frankfurt type	79
NPYR (μg/day)	0.03 (0.01 – 0.06)	Cooked ham, extra; chorizo, category w/s; black pudding; cured ham, lean and fat; sausage, Frankfurt type	79
**Acrylamide (μ g/day**)	8.73 (6.44 – 11.62)	Cookie; potato; white bread, loaf; wholemale bread, loaf; white bread, stick	91
**(Poly)phenols (mg/day)**			
Total (poly)phenols	683.17 (345.80 – 1208.90)	Coffee, infusion; potato; lentils; soluble cocoa powder; banana	62
Flavonoids	62.37 (10.71 – 183.03)	Onion; orange juice, commercial; orange juice, fresh; cherry; orange	52
Phenolic acids	226.37 (101.87– 605.21)	Coffee, infusion; potato; green olive, in brine; cherry; carrot	93
Lignans	12.98 (8.73 – 27.08)	Potato; green bean; tomato; melon; carrot	59
Other (poly)phenols	13.74 (4.84 – 23.24)	Coffee, infusion; olive oil, virgin; green olive, in brine; pasta; olive oil	88
Stilbenes	0.00 (00.00 – 0.02)	Lentil; vinegar; green grape; peanut butter; red wine	48
**Dietary fiber (g/day)**			
Total	11.86 (8.29 – 15.06)	White bread, stick; potato; pasta; chick peas; white bread, loaf	35
Soluble	1.46 (1.05 – 2.07)	White bread, stick; potato; pasta; white bread, loaf; tomato	50
Insoluble	5.97 (4.56 – 10.25)	White bread, stick; potato; pasta; wholemale bread, loaf; onion	43
Starch	23.31 (9.07 – 42.44)	Pasta; breadcrumbs; pizza, tomato and cheese, baked; white bread, loaf; wheat flour	87
Celulose	2.15 (1.74 – 3.37)	Potato; white bread, stick; pasta; lentils; onion	43
Klason Lignine	1.13 (0.71 – 1.49)	White bread, stick; pasta; white bread, loaf; banana; wholemale bread, loaf	56
Hemicelulose			
Soluble	1.14 (0.48 – 1.36)	White bread, stick; pasta; potato; white bread, loaf; cookie	63
Insoluble	2.12 (1.51 – 3.62)	Pasta; potato; white bread, stick; wholemale bread, loaf; onion	49
Pectin		White bread, stick; potato; pasta; chick peas; white bread, loaf	
Soluble	0.45 (0.26 – 0.62)	Potato; banana; tomato; onion; carrot	58
Insoluble	0.71 (0.45 – 1.18)	Potato; onion; lettuce; green bean; carrot	50

Data is expressed as median (P_25_ – P_75_). For each dietary compound five major food dietary sources and mean percentage of contribution (%) to the total intake in the sample are shown. MeIQx, 2-amino-3,8 dimethylimidazo (4,5,f) quinoxaline; DiMeIQx, 2-amino-3,4,8 trimethylimidazo (4,5,f) quinoxaline; PhIP, 2-amino-1-methyl-6-phenylimidazo (4,5,b) pyridine; B(a)P, benzo (a) pyrene; DiB(a)A, dibenzo (a) anthracene; Total PAH, total polycyclic aromatic hydrocarbons; NDMA, N-nitrosodimethylamine; NPIP, N-Nitrosopiperidine; NPYR, N-Nitrosopyrrolidine.

### Gut microbiota profile

**FIGURE 1:**
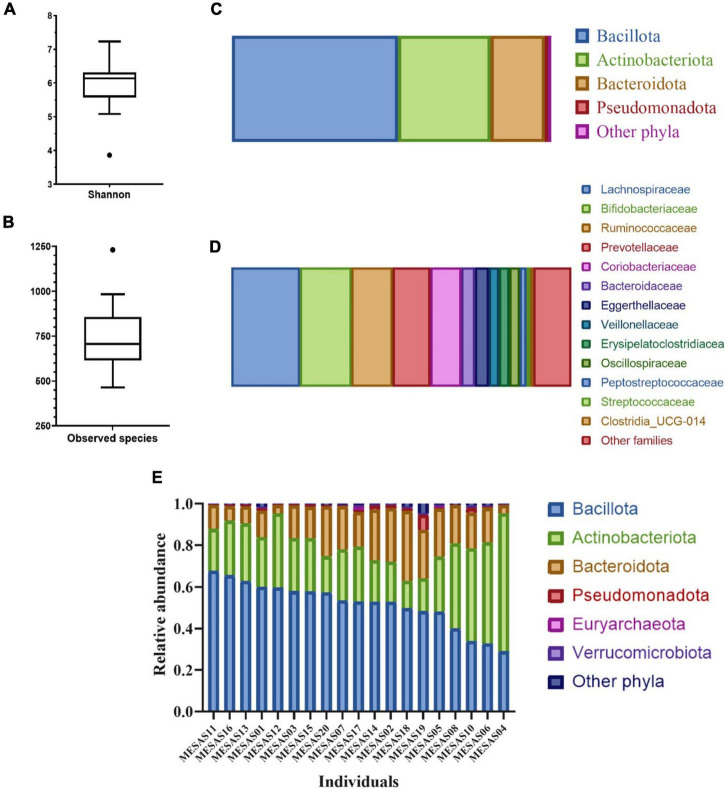
Diversity indices and GM relative abundance composition. Box plot of Shannon index **(A)** and Observed species index **(B)**. GM profile at phylum **(C)** and family level **(D)**; only taxa showing mean relative abundances higher than 1% are shown. GM main phyla relative abundance distribution in the sample of study disaggregated by individual **(E)**.

The GM diversity determined by the Shannon index and richness measured as Observed species were 6.13 and 706, respectively (Figures 1A,B). At the phylum level, Bacillota was the most abundant, followed by Actinobacteriota, Bacteroidota, and Pseudomonadota (Figure 1C). At the family level, the most abundant was Lachnospiraceae, followed by Bifidobacteriaceae, Ruminococcaceae, Prevotellaceae and then Coriobacteriaceae (Figure 1D). GM relative abundances at the phylum level, disaggregated by individual evidenced a global increase of Bacteroidota at the expenses of the decrease of Bacillota from individuals MESAS11 to MESAS19, (with the exception of individual MESAS12) in contrast to an increase of Actinobacteriota at the expenses of the reduction of Bacillota in the remaining individuals (Figure 1E).

### Dietary patterns and gut microbiota

In order to look for dietary, anthropometric, biochemical or lifestyle factors that could be related with variations in the two most abundant intestinal microbial phyla (Bacillota and Actinobacteriota) in the sample, individuals were clustered in two groups according to the Bacillota/Actinobacteriota abundance ratio: group 1 (ratio ≥2) and group 2 (ratio <2). Individuals in the group 2 presented a significantly higher consumption of red meat and significantly higher fasting glucose levels as well as a total/HDL-cholesterol atherogenic index higher than individuals from the group 1, without reaching risk threshold values of 110 mg/dL and 4.5 defined for these blood parameters, respectively (Table 4). When comparing the GM between both groups of individuals, several taxa displayed significant differences (Table 4). Remarkably, the only significant variation within the Actinobacteriota phylum was found for the family Bifidobacteriaceae and the genus *Bifidobacterium*, which had twice the relative abundances in the group that consumed more red meat than individuals who consumed less red meat. No other anthropometric, biochemical, lifestyle or GM parameters displayed significant differences between these two subgroups in the sample (data not shown).

**TABLE 4:** Dietary and microbiota differences according to microbiota profile distribution of main phyla in the sample of study.

Characteristics	Group 1 (*N* = 13)	Group 2 (*N* = 6)
***Dietary***		
Red meat (g/day)	21.00 ± 21.89	70.20 ± 36.65*
***Biochemical parameters***		
Fasting glucose (mg/dL)	85.31 ± 7.84	95.50 ± 5.32*
Total cholesterol/HDL ratio	3.49 ± 0.73	4.78 ± 1.31*
***Microbiota composition***		
Bacillota	56.85 ± 5.91	40.57 ± 11.51*
Lachnospiraceae	22.42 ± 4.66	14.58 ± 3.66*
*Agathobacter*	6.39 ± 1.61	3.41 ± 1.96*
*Blautia*	3.77 ± 1.40	2.75 ± 1.24*
*Roseburia*	1.21 ± 0.90	0.49 ± 0.04*
Ruminococcaceae	13.27 ± 2.99	9.01 ± 0.98*
*Faecalibacterium*	5.54 ± 1.44	3.38 ± 0.89*
*Ruminococcus torques* group	1.33 ± 0.81	0.63 ± 0.17*
Peptostreptococcaceae	2.84 ± 1.70	1.46 ± 0.51*
*Romboutsia*	2.26 ± 1.30	1.16 ± 0.41*
Actinobacteriota	21.88 ± 4.67	43.67 ± 13.49*
Bifidobacteriaceae	9.41 ± 4.02	26.95 ± 19.31*
*Bifidobacterium*	9.40 ± 4.02	26.94 ± 19.31*
Pseudomonadota	1.44 ± 1.82	0.60 ± 0.81*

Data is expressed as mean ± standard deviation. For microbiota composition relative abundance (%) for those taxa >1% are shown.

(*) Only significant results found by the U-Mann Whitney test *p*-value <0.05 are displayed on the table.

HDL, high density lipoprotein.

### Xenobiotics, (poly)phenols, dietary fibers, and gut microbiota

**FIGURE 2:**
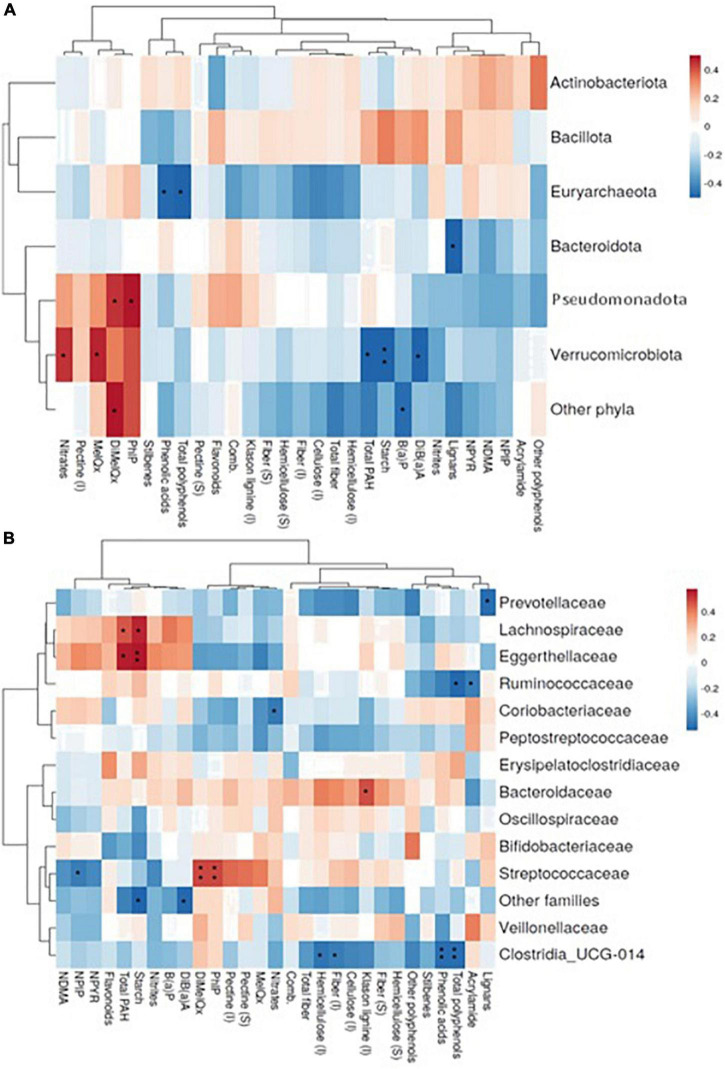
Spearman correlations representation between the most abundant bacterial phyla **(A)** or families **(B)** (rows) and consumption of xenobiotics and bioactive compounds derived from the diet in the sample (columns). (*), (**) *p* – value < 0.05 and 0.01, respectively. Only taxa showing mean relative abundances higher than 1% are shown. MeIQx, 2-amino-3,8 dimethylimidazo (4,5,f) quinoxaline; DiMeIQx, 2-amino-3,4,8 trimethylimidazo (4,5,f) quinoxaline; PhIP, 2-amino-1-methyl-6-phenylimidazo (4,5,b) pyridine; B(a)P, benzo (a) pyrene; DiB(a)A, dibenzo (a) anthracene; Total PAH, total polycyclic aromatic hydrocarbons; NDMA, N-nitrosodimethylamine; NPIP, N-Nitrosopiperidine; NPYR, N-Nitrosopyrrolidine; (I), insoluble; (S), soluble.

Associations of dietary components with GM are shown at the phylum (Figure 2A) and family level (Figure 2B). Pseudomonadota and Verrucomicrobiota are the phyla showing the most significant direct correlations with xenobiotics (HAs, total PAHs, and nitrates). Verrucomicrobiota is also significantly associated with some fibers and (poly)phenols, as well as Euryarchaeota and Bacteroidota. These phyla are inversely correlated with starch, (poly)phenols and phenolic acids and lignans, respectively. At the family level (Figure 2B), 2-amino-3,4,8 trimethylimidazo (4,5,f) quinoxaline (DiMeIQx) and 2-amino-1-methyl-6-phenylimidazo (4,5,b) pyridine (PhIP) are directly associated with Streptococcaceae, this family being inversely correlated with the nitrosamine N-Nitrosopiperidine (NPIP). From (poly)phenols, inverse associations were detected for some of them with Clostridia UCG-014, Prevotellaceae and Ruminococcaceae while starch is directly associated with Lachnospiraceae and Eggerthellaceae. Based on these results, a stepwise regression analysis was conducted for the major microbial phyla and dietary sources, potential carcinogens, and (poly)phenol, controlled by age, BMI and energy intake (Table 5). The results obtained revealed that the associations represented in Figure 2 remained after adjusting for energy intake and BMI. It is also remarkable that in most cases dietary sources are not the main variables explaining the correlations found but the specific dietary component instead is sufficient by itself to explain at least for the minimum coefficient of the association.

**TABLE 5:** Analysis of variables accounting for significant correlations found between microbiota and the intake of xenobiotics, fibers and (poly)phenols, at the phylum level.

		Variables	*R* ^2^	β	*p*
**Bacteroidota**					
	Model 1	Lignans	0.302	−0.584	0.009
	Model 2	Lignans Apricot	0.428	−0.754 0.425	0.001 0.044
	Model 3	Lignans Apricot Age	0.626	−1.054 0.508 0.523	0.000 0.006 0.008
	Model 4	Lignans Apricot Age Pear	0.778	−0.915 1.253 0.635 −0.915	0.000 0.000 0.000 0.005
	Model 5	Lignans Apricot Age Pear Melon	0.829	−0.893 1.225 0.564 −0.920 −0.232	0.000 0.000 0.001 0.002 0.041
**Pseudomonadota**					
	Model 1	Chicken, thigh, skinless	0.238	0.529	0.020
	Model 2	Chicken, thigh, skinless PhIP	0.423	0.462 0.459	0.021 0.022
**Verrucomicrobiota**					
	Model 1	MeIQx	0.349	0.621	0.005
	Model 2	MeIQx Pasta	0.541	0.591 −0.456	0.002 0.012
**Other phyla**					
	Model 1	DiMeIQx	0.191	0.486	0.035

Results from stepwise regression analysis between significant correlated associations of main phyla relative abundances and the intake of dietary compounds (xenobiotics, fibers and (poly)phenols). The variables considered for each analysis are BMI, age, energy intake and ALL dietary sources involved in each case. Only significant results are shown. β, standarized regression coefficient; *R*^2^, adjusted coefficient of multiple determination; *p*; *p* value. MeIQx, 2-amino-3,8 dimethylimidazo (4,5,f) quinoxaline; DiMeIQx, 2-amino-3,4,8 trimethylimidazo (4,5,f) quinoxaline; PhIP, 2-amino-1-methyl-6-phenylimidazo (4,5,b) pyridine.

### Analysis of the gut microbiota according to the risk for xenobiotic intake levels

**FIGURE 3:**
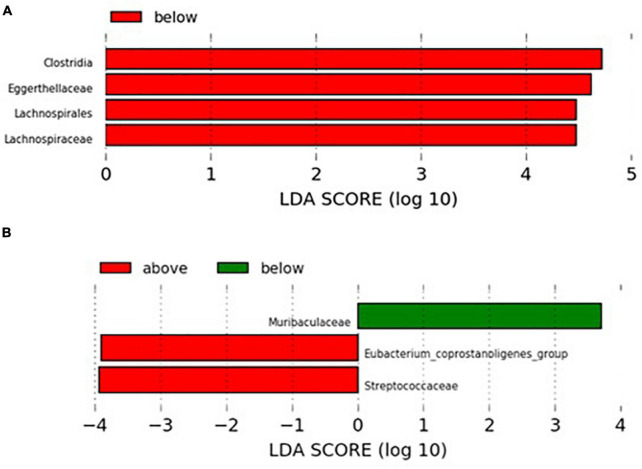
LEfSe analysis of microbial taxa differentially altered as a function of safety intake thresholds for some xenobiotics. **(A)** MeIQx at levels below (*n* = 11) or over (*n* = 8) the risk dose (50 ng/day) (54) and **(B)** PhIP at levels below (*n* = 12) or over (*n* = 7) the risk dose (40 ng/day) (54). Only families with relative abundance of at least 1% in at least two samples were considered in the analysis.

Since xenobiotic intake can vary widely between individuals, a LEfSe analysis was conducted to detect differences in GM profiles between individuals consuming xenobiotics below or over the risk doses described in the literature. PhIP and MeIQx were the only carcinogenic compounds showing significant associations with specific taxa of the GM both by Spearman correlation and by stepwise regression analysis for which risk daily consumption doses were reached in the sample. In the case of MeIQx (Figure 3A), higher levels of Lachnospiraceae and Eggerthellaceae families were found in individuals consuming less than 50 ng/day (lower risk for colorectal adenoma) (54) while those consuming more than 40 ng/day of PhIP (higher risk for colorectal adenoma) (54) showed lower relative abundance of Muribaculaceae and greater presence of Streptococcaceae and *Eubacterium coprostanoligenes* group (Figure 3B).

## Discussion

In developed countries, foods with solid science-based evidence on their benefits for health, such as fruit, vegetables, whole grains, and fish, usually have a relatively high cost. As a result, vulnerable at-risk groups often adopt diets that are far from the Mediterranean dietary standard (9), increasing the risk long-term to suffer non-communicable diseases, such as diabetes, hypertension, hyperlipidemia and obesity (55, 56). To the best of our knowledge, this is the first study examining the potential impact of the diet of a socially vulnerable population on the composition of the GM, providing a more in-depth analysis into the HAs, PAHs, nitrates, nitrites, nitroso compounds and acrylamide resulting from food processing. In spite of this, it is interesting to note that in our sample those individuals consuming significantly higher amounts of red meat also displayed significantly higher levels of microorganisms from the genus *Bifidobacterium* than individuals with lower intake of meat. Members of the genus *Bifidobacterium* are considered beneficial for health (57). Lower intestinal *Bifidobacterium* levels have been generally associated with higher consumption of red meat and animal meat (58) in the context of high fat and high calorie westernized diets, with high consumption of meat. However, the population analyzed in the present study are socially disadvantaged individuals whose daily intake of red meat did not exceeded the maximum recommended (53). In this regard, lower levels of fecal *Bifidobacterium* have been also reported in vegetarian individuals with respect to omnivores (59).

Our results evidence that the intake of some (poly)phenol, fibers and xenobiotics derived from food processing were associated with the GM composition, with a differential impact as depending on the microbial groups. It seems plausible that the consumption of processed foods, as well as fast cooking techniques, may lead to a higher intake of carcinogens in this sample. In this regard, only 42% and 37% of the sample had a MeIQx or PhIP intake above the recommended values (50 ng/day and 40 ng/day, respectively) (54). The average daily intakes of nitrate (54 mg) and acrylamide (10 μg) were within the normal limits, with no volunteers exceeding threshold levels (3.70 and 0.17 mg/kg/day, respectively) (60, 61). When comparing the levels of xenobiotics intake obtained in the present work with those of other studies in the general population at the same geographical location, a higher consumption of MeIQx and lower of phenolic compounds and fibers was observed in our sample (62). This is consistent with the lower consumption of fruit, vegetables, and plant-based foods in this human group.

Net effects exerted by dietary xenobiotics on the GM are dependent on their intake levels, the diet considered globally, and the interactions between these compounds and the GM (18). Indeed, microorganisms from the GM can present different degrees of sensitivity/resistance to dietary xenobiotics. Some members of the GM can bind toxics, contributing to their elimination with feces, and others can metabolize toxics, directly or through microbial metabolic interactions, resulting in new derived compounds with higher or lower toxicity. In this way, some of the variations in relative abundances of gut microbial taxa studied in the present work may be related with the microbial fitness exhibited against some of the potential carcinogens, fibers and (poly)phenols. The effect of each toxic compound could be enhanced or attenuated by other dietary components, which can result in associations of xenobiotics and/or bioactive compounds with the GM that vary depending on the characteristics of the GM itself, dietary habits and lifestyle. Therefore, although causality cannot be established from this type of research, it is necessary to highlight the difficulty in evaluating the impact of dietary components in human populations. In this sense, food groups, such as vegetables, which are sources of nitrites but also of (poly)phenols and fiber may have a different association with the GM than those compounds derived from cooked meat. For some dietary phenolics and fibers it has been demonstrated experimentally that they can counteract, total or partially, the effects of potentially harmful xenobiotics, even avoiding their formation during cooking, thus decreasing the potential toxicity of foreign chemicals to the organism (63–65). These associations between the GM and xenobiotics have been demonstrated by *in vitro* and *in vivo* systems. For instance, the formation of quinone-derived compounds was prevented from (poly)phenols of green tea in the presence of N-nitrosamine by the action of gut microbiome (66). Likewise, dietary wheat bran was shown to attenuate chronic cadmium toxicity in rats (67) and mulberry and dandelion water extracts were shown to prevent alcohol-induced steatosis and alleviate gut microbiome dysbiosis in rats (68). However, as our sample presented an intake of the HA MeIQx higher than the recommended levels and a low intake of phenolic compounds, the potential protective effect of dietary (poly)phenols would be presumably lower than in a population with higher intake of fruits and vegetables. Nevertheless, the long-term effect of the interaction between dietary bioactive compounds such as (poly)phenols and fibers from vegetables and xenobiotics derived from food processing, as HAs and PAHs, at the intestinal level and their effect on the GM remains largely unknown.

At the phylum taxonomic level, associations of several xenobiotics (mainly HAs) with the GM seem to have an opposite direction to that of several (poly)phenols whereas other potentially harmful xenobiotics display associations similar to dietary compounds derived from plants. This is the case of total PAH, dibenzo (a) anthracene (DiB(a)A) and starch, all of them showing negative association with Verrucomicrobiota and Euryarchaeota and positive correlation with the phylum Bacillota. In addition, both Pseudomonadota and Verrucomicrobiota are directly correlated with some HAs (MeIQx, DiMeIQx, and PhIP) and nitrates. Verrucomicrobiota is also significantly associated with some bioactive dietary components, as well as Euryarchaeota and Bacteroidota. Thus, these three phyla (and the family Prevotellaceae within the phylum Bacteroidota) are inversely correlated with starch, (poly)phenols and phenolic acids, and lignans, respectively. It has been generally reported that the intake of dietary sources rich in (poly)phenols and/or fiber can shape the GM by promoting the abundance of beneficial bacteria and inhibiting some pathogenic microbial groups (69–71) whereas food chemicals can disrupt human GM and impact negatively the intestinal homeostasis (72). Studies focusing on the association between xenobiotics and GM are still scarce and data available are mainly from *in vitro* models and *in vivo* murine models. In this regard, Kim and Hur (73) found during *in vitro* simulated human digestion, that the mutagenicity of HAs was drastically reduced in the presence of enterobacteria, *Escherichia coli* and *Lactobacillus sakei*. Ribière et al. (74) evidenced in a murine model that oral exposure to B(a)P induced an increase in the relative abundance of Bacteroidaceae, Porphyromonadaceae and Paraprevotellaceae and decreased Lactobacillaceae and Verrucomicrobioaceae families. Furthermore, the genus *Bifidobacterium* and families Coriobacteriaceae, Rikenellaceae and Desulfovibrionaceae increased in the presence of this xenobiotic derived from food processing.

Consistent with some of the associations found for HAs and the most abundant intestinal microbial taxa, we evidenced differentially higher abundance of the genus *Streptococcus* and members of the *Eubacterium coprostanoligenes* group in the GM of individuals with daily intake of PhIP in doses considered at higher risk. In contrast, individuals with lower intake of this xenobiotic displayed differentially higher abundance of the family Muribaculaceae. Differentially higher abundance of Eggerthellaceae and Lachnospiraceae was found in those individuals with daily intake of MeIQx below the doses considered at risk. *Eubacterium coprostanoligenes* has been related with the maintenance of intestinal mucosal barrier function (75), so that their differentially higher levels in individuals with higher intake of PhIP may be interpreted as a reinforcement of the protection of the intestinal mucosa against moderately high intake of this xenobiotic. In contrast, Lachnospiraceae tends to be differentially reduced in pathological states (76–78) whereas Muribaculaceae, a recently described family, has been related with long-term health effects (79, 80).

Interpreting the findings on the relationship between xenobiotics and GM obtained in our human sample is challenging. The scientific literature currently available generally describes positive association of the genus *Akkermansia* (phylum Verrucomicrobiota) with dietary resistant starch (81), which is apparently contradictory with our results. These differences could be partially related to the inverse relationship between the levels of Clostridia and Lachnospirales (Bacillota phylum) and Eggerthellaceae (Actinobacteriota phylum) and MeIQx intake, as well as to the existence of some key species in the degradation of resistant starch, such as *Ruminococcus bromii* (82). The previously reported negative association between resveratrol with the intestinal microbial genus *Methanobrevibacter* (Euryarchaeota phylum) in humans is according to our results and could be related with the complex crosstalk among the (poly)phenols consumption, intestinal permeability and GM composition (83). The inverse association between the relative abundance of Prevotellaceae (phylum Bacteroidota) and lignans could be related with some other positive association found by us at the family level for other members of the GM with lignans, as it is the case of Peptostreptococcaceae and Coriobacteriaceae (genus *Eggerthella*), two groups of microorganisms with strains able to participate in the metabolization of lignans (26). Other positive and negative associations found in the present work between xenobiotics (toxic and bioactive compounds) and GM may be due to changes in the relative abundance of the different microbial taxa at a variable extent, depending on their interaction with dietary xenobiotics. Our work suggests a comparatively higher potential carcinogens exposure and a lower consumption of protective bioactive compounds in the healthy vulnerable population under study with respect to other groups at the same geographical location (62). This was accompanied by differential intestinal microbial altered profiles in those individuals with intake of certain xenobiotics over the risk threshold, which can potentially increase the risk of long-term non-communicable diseases. Comparing dietary habits of African American volunteers (a population presenting increased incidence and mortality by colorectal cancer) and Caucasian Americans evidenced higher intake of HAs and decreased intake of vitamins, including vitamin D in the first group, which was correlated with differences in GM composition (84). Groups of economically and socially vulnerable individuals may be susceptible for early basic nutritional interventions to improve their nutritional and GM profile if these results will be confirmed in future studies.

## Conclusion

The results obtained point to a possible association between potential carcinogens in the diet and the composition of the GM in subjects with a low socioeconomic level, despite the limited sample size of this work. However, when extrapolating the results, it should be taken into account the proportion of gender in the sample and the high BMI, both factors that could influence the composition of the GM. If confirmed in future studies, these data would serve to evidence the need for strategies aimed at nutritional intervention in these groups for the promotion of health.

## Data availability statement

The raw data presented in the study are deposited in the NCBI BioProject repository, accession number: PRJNA870886.

## Ethics statement

The studies involving human participants were reviewed and approved by Ethics Committee of the Hospital Universitario Central de Asturias (CEImPA2021.307). The patients/participants provided their written informed consent to participate in this study.

## Author contributions

SG and CR-G designed the study. SG and AZ recruited the participants. AZ performed the nutritional assessment and statistical analysis. SA, SR-S, MG-M, NS, AN, and MG contributed and assisted to methodology and analytical tools. SG, CR-G, and AZ drafted the manuscript. All authors have read and agreed to the published version of the manuscript.
